# Novel *in Vitro* Model for Keratoconus Disease

**DOI:** 10.3390/jfb3040760

**Published:** 2012-11-13

**Authors:** Dimitrios Karamichos, Ramin Zareian, Xiaoqing Guo, Audrey E.K. Hutcheon, Jeffrey W. Ruberti, James D. Zieske

**Affiliations:** 1 Schepens Eye Research Institute/Massachusetts Eye and Ear, Department of Ophthalmology, Harvard Medical School, Boston, MA 02114, USA; Email: xiaoqing.guo@schepens.harvard.edu (X.G.); audrey.hutcheon@schepens.harvard.edu (A.H.); james.zieske@schepens.harvard.edu (J.Z.); 2 Mechanical & Industrial Engineering, Northeastern University, Boston, MA 02115, USA; Email: zareian.r@husky.neu.edu (R.Z.); j.ruberti@neu.edu (J.W.R.)

**Keywords:** Keratoconus disease, extracellular matrix, TGF-β3, fast fourier analysis, corneal fibroblasts

## Abstract

Keratoconus is a disease where the cornea becomes cone-like due to structural thinning and ultimately leads to compromised corneal integrity and loss of vision. Currently, the therapeutic options are corrective lenses for early stages and surgery for advanced cases with no *in vitro* model available. In this study, we used human corneal fibroblasts (HCFs) and compared them to human Keratoconus fibroblasts (HKCs) cultured in a 3-dimensional (3D) model, in order to compare the expression and secretion of specific extracellular matrix (ECM) components. For four weeks, the cells were stimulated with a stable Vitamin C (VitC) derivative ± TGF-β1 or TGF-β3 (T1 and T3, respectively). After four weeks, HKCs stimulated with T1 and T3 were significantly thicker compared with Control (VitC only); however, HCF constructs were significantly thicker than HKCs under all conditions. Both cell types secreted copious amounts of type I and V collagens in their assembled, aligned collagen fibrils, which increased in the degree of alignment upon T3 stimulation. In contrast, only HKCs expressed high levels of corneal scarring markers, such as type III collagen, which was dramatically reduced with T3. HKCs expressed α-smooth muscle actin (SMA) under all conditions in contrast to HCFs, where T3 minimized SMA expression. Fast Fourier transform (FFT) data indicated that HKCs were more aligned when compared to HCFs, independent of treatments; however, HKC’s ECM showed the least degree of rotation. HKCs also secreted the most aligned type I collagen under T3 treatment, when compared to any condition and cell type. Overall, our model for Keratoconus disease studies is the first 3D *in vitro* tissue engineered model that can mimic the Keratoconus disease *in vivo* and may be a breakthrough in efforts to understand the progression of this disease.

## 1. Introduction

Keratoconus was first described in 1748 by the German oculist, Burchard Mauchart, who provided an early description of the disorder [1], which he called *staphyloma diaphanum*. It was not until 1854, however, that Keratoconus was clearly described and distinguished from other corneal defects by the British physician, John Nottingham [2]. Prevalence of the disease has been reported ranging from 4 to 600 in 100,000 people [3,4,5,6].

Keratoconus can appear as early as puberty and progress at various speeds for decades [4]. Clinically, early detection of the disease is uncommon, mainly because the signs and symptoms are often confused with severe astigmatism cases. Normally, the disease has to progress before it can be detected. To date, there are several treatments available, depending upon severity. For early stages, the most common treatment is the use of hard contact lenses known as rigid gas-permeable, or RGPs, which provide a good level of visual correction, but do not slow down or stop the progression [3]. For more advanced cases, surgery—corneal transplantation, deep lamellar keratoplasty or corneal ring segment inserts—is inevitable [3,7]. Vision is normally improved after surgery, however, possible complications may be related to vascularization of the corneal tissue and rejection of the donor cornea [4]. 

Despite considerable research, the etiology of Keratoconus remains unclear [1]. In the epithelium, the basal layer normally degenerates and breaks down leading to the dissolution of the Bowman’s layer [4,8]. As the epithelium and the stromal layers come closer to each other, structural and cellular alterations occur leading to scarring, corneal thinning and bulging [3,4,8]. In the stroma, fibrils are compacted and lose their arrangement. Also, normal fibroblasts, keratocytes and collagen lamellae numbers decrease; however, the endothelium is normally unaffected.

The aim of this study was to examine HKC in a 3D *in vitro* model. These studies revealed that HKC in this system might provide a model for the investigation of Keratoconus disease. To the author’s knowledge, this is the first 3D *in vitro* model available which can characterize and stimulate Keratoconus cells within their own secreted ECM. Furthermore, we were able to partially “rescue” the HKC-phenotype so that it secreted a matrix that was closer to normal stromal ECM. Further studies are currently ongoing. The development of such a novel model may provide clues for development of novel therapeutics to treat Keratoconus.

## 2. Methods

### 2.1. Primary Culture of Human Keratoconus (HKC) and Human Corneal Fibroblast Cells (HCF)

HKCs were isolated from human corneas from patients with Keratoconus defects. These corneas were obtained from Ula Jurkunas (Massachusetts Eye and Ear Infirmary, Boston, MA, USA). HCFs were isolated from normal human corneas obtained from NDRI (National Disease Research Interchange; Philadelphia, PA). Briefly, corneal epithelium and endothelium were removed from the stroma by scraping with a razor blade. The stromal tissue was cut into small pieces (~ 2 × 2mm) and put into 6-well plates (4 or 5 pieces per well). Explants were allowed to adhere to the bottom of the wells and Eagle’s Minimum Essential Medium (EMEM: ATCC; Manassas, VA) containing 10% fetal bovine serum (FBS: ATCC) was added. After 1–2 weeks of cultivation, the cells were passaged into a 100 mm cell culture plate. The cells were allowed to grow to 100% confluence before being used in the culture system. Morphologically, HKCs exhibited characteristic myofibroblastic morphology, such as prominent cytoplasmic actin microfilaments (stress fibers) and high levels of α-smooth muscle actin (SMA) expression (data not shown), that distinguished them from healthy HCFs.

### 2.2. Assembly of Extracellular Matrix

Both HCFs and HKCs were plated on transwell 6-well plates containing polycarbonate membrane inserts with 0.4 μm pores (Costar, Charlotte, NC, USA,) at a density of 10^6^ cells/mL. The protocol followed was identical for both cell types. Cells were cultured in EMEM with 10% FBS and 0.5 mM 2-O-α-D-glucopyranosyl-L-ascorbic acid (VitC, Wako Chemicals USA, Inc.; Richmond, VA, USA). The cultures were allowed to grow for 4 weeks in the presence of 0.1ng/mL TGF-β1 (T1) or TGF-β3 (T3). Cultures without any growth factors served as Controls (C). Culture medium was changed every other day for the duration of the experiment. The morphology of the cultures was examined using transmission electron microscopy (TEM). In addition, indirect-immunofluorescence was used to identify specific markers of stromal components—Smooth muscle actin (SMA), type I collagen (Col I), type III collagen (Col III), type V collagen (Col V) and fibronectin (EDA-Fn). 

### 2.3. Indirect-Immunofluorescence (IF)

After 4 weeks in culture, constructs were collected and fixed in 4% paraformaldehyde before being processed for IF, as previously described [9]. Briefly, each sample was incubated at 4 °C overnight with the following primary antibodies diluted in 1% BSA + 0.1% Triton-X: anti-Col I, III, and V (Southern Biotech, Birmingham, AL, USA), anti-EDA-Fn (Sigma Aldrich, St. Louis, MO, USA), and anti-SMA (Dako North America, Carpinteria, CA, USA). The samples were then washed and incubated overnight at 4 °C with the corresponding secondary antibody in 1% BSA + 0.1% Triton-X—donkey anti-goat (Col III), anti-mouse IgM (EDA-Fn), and anti-mouse IgG (SMA, Col I and V). TO-PRO-3 iodide (Life Technologies Corporation, Grand Island, NY, USA) was used as a marker of cell nuclei. Finally, constructs were washed, mounted with Vectashield Mounting Media (Vector Laboratories, Inc.; Burlingame, CA, USA), and then observed and photographed using a confocal TCS-SP2 or TCS-SP5 Leica microscope (Leica Microsystems; Bannockburn, IL, USA). For negative controls, the primary antibody was omitted.

At this time, the construct’s thickness was also measured as previously described [10]. Briefly, using the confocal microscope’s z-scans, measurements began at the top of the construct (first cell visible) and ended at the bottom of the construct (last cell visible). Thicknesses were analyzed (7–11 samples per condition) for significant difference (p < 0.05)

### 2.4.Transmission Electron Microscopy (TEM)

At week 4, constructs were collected and fixed in ½ strength Karnovsky’s fixative at 4 ºC overnight. After which, they were processed for TEM, as described in Gipson *et al*. [11]. Briefly, constructs were rinsed in PBS, processed through post-fixation in 2% osmium tetroxide, en bloc stained in 0.5% uranyl oxide, dehydrated with alcohol to propylene oxide, and embedded in Embed 812 (Electron Microscopy Sciences; Hatfield, PA, USA). Thin sections, transverse to the plane of the construct, were cut with a diamond knife on an ultramicrotome (LKB, Bromma, Sweden). The sections were viewed and photographed with a Tecnai G^2^ Spirit (FEI Company, Hillsboro, OR, USA). On average, 20 photos were used per condition per cell type. All experiments were repeated at least three times.

### 2.5. Fast Fourier Transform Analysis (FFT)

FFT is a method to describe the anisotropy of fibers in binary images by using image processing. In this study, we took the images collected from the confocal microscope and quantified the orientation of the collagen fibers and cells at each z-section by applying a graphical FFT. The graphical FFT uses spectral techniques with the aid of 2-dimensional (2D) FFT in the form of vectors in order to graphically quantify the orientation of the fibers or cells [12,13]. Briefly, confocal z-series stack images were converted to grayscale images and the distribution of fibers or cells were captured using our custom MATLAB software. The distribution of orientations was shown on polar plots where the intensity of the 2D FFT profiles was converted into polar coordinates. In each case, the median and the range of the orientations from the top 70% of the population were reported as indicators of the main orientation and extent of alignment at that orientation respectively. Therefore, wider histograms represent a higher range of alignment, which corresponds to more randomly oriented fibers or cells in an image. In order to obtain the values for rotation, the change in the angle of orientation between z-scans were calculated and applied.

### 2.6. 3D Cell Counting Software

Image Pro Plus (Image Pro Plus v.7: Media Cybernetics, Bethesda, MD, USA) is a 2 and 3D analysis software program that was used to analyze and quantitate data from the confocal z-series, as previously described [14]. Briefly, Image Pro Plus was used to count the number of cells in each section (plane-of-focus) of a confocal z-series in order to quantitate the number of cells per construct. At least three confocal z-series were quantitated per condition and their average was analyzed.

### 2.7.Statistical Analysis

All experiments were repeated at least three times and data was analyzed for significant variations (*p* < 0.05) using the Student’s t-test and Dunnett’s Multiple Comparison test.

## 3. Results

### 3.1. Constructs Development

In our previous work [14,15], we have shown that different cell types, such as HCF and umbilical cord stem cells, have the ability to secrete and lay down a fibrous collagen ECM in the presence of VitC. We have particularly demonstrated the effect of T1 and T3 in these cultures [14]. In the current study, we examined the ability of HKCs to synthesize a matrix in the presence of VitC ± T1 or T3. Matrix thinning is one of the end results in patients with Keratoconus disease; therefore, the study for matrix secretion and deposition is critical. 

**Figure 1:**
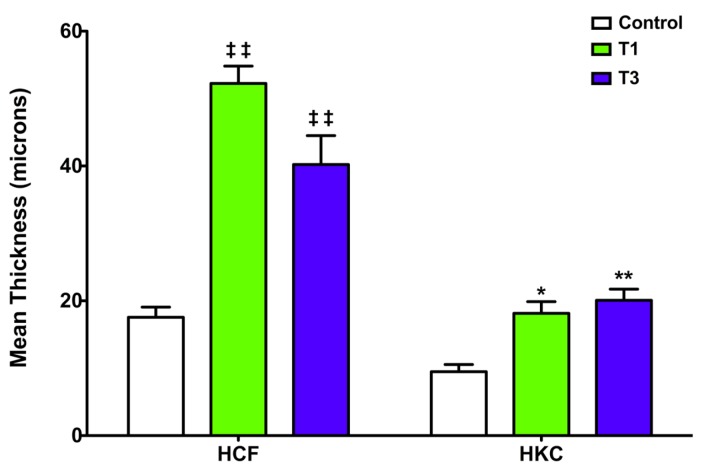
Mean construct thickness of human corneal fibroblasts (HCFs) and human Keratoconus fibroblasts (HKCs) treated with VitC only (Control), VitC + TGF-β1 (T1) or VitC + TGF-β3 (T3). T1 and T3 treatments led to an increase in construct thickness for both the HCFs (‡‡ = p<0.001) and the HKCs (* = p < 0.05 and ** = p < 0.001). Note that the HCFs total thickness was higher when compared to HKCs; however, when HKCs were treated with either T1 or T3, the thickness was similar to HCF control.

After 4 weeks in VitC only, the HKCs’ construct had a mean thickness of 9.5 μm, whereas, the HCFs synthesized a matrix of 17.6 μm thick (Figure 1). When T1 was added to the cultures, the HKC construct’s thickness increased significantly (18.2 μm: p < 0.05; Figure 1); however, this is still less than the HCFs’ construct, where the thickness increased to 52.3 μm (*p* < 0.001; Figure 1). Upon T3 stimulation, the HKC and the HCF constructs maintained similar thicknesses as with T1 (20.1 μm and 40.2 μm, respectively). Note that with all the conditions, the HKCs secreted significantly less matrix when compared to the HCFs (p < 0.05); however, with the addition of T1 or T3, the HKCs were of comparable thickness as the HCF VitC only constructs. This data suggests that HKCs can be stimulated to produce more matrix, thus partially “rescuing” the HKCs to be more like HCFs.

In order to determine whether this increase in thickness was the result of an increase in the number of cells or an increase in the amount of matrix produced per cell, we examined the total cell number for each construct under all conditions. Results for both cell types showed a 2-3-fold increase in cell number following TGF-β stimulation compared to controls (data not shown). However, the amount of ECM produced per cell remained relatively constant under all conditions.

### 3.2. Ultrastructural Characterization

**Figure 2:**
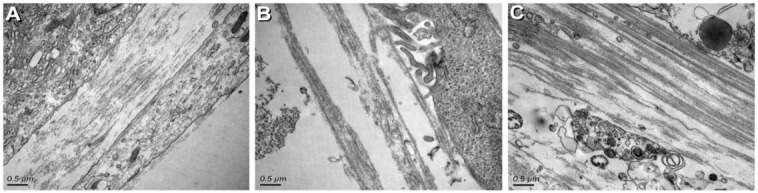
High magnification TEM (31,000x) of HKC constructs showing cell matrix interaction and matrix condition: (**A**) Control (VitC only); (**B**) TGF-β1 treated (T1) and (**C**) TGF-β3 treated (T3). Note that the T3-treated construct (C) has a higher density of collagen fibrils and ECM alignment, compared to controls (A) and T1 (B). Bars = 0.5 microns.

TEM was used to observe the cell-ECM interaction in each of the HKC constructs at the end of 4 weeks. HCFs were used for comparison (data not shown) [10]. As seen in Figure 2A,B (Control and T1, respectively), areas of organized ECM were apparent, and there was a higher density of collagen fibrils and ECM alignment in the control constructs (Figure 2A) than the T1 (Figure 2B). This data is opposite to that of the HCF, where upon the addition of T1 to the HCF constructs, the collagen fibrils were more organized and compact than in control (data not shown) [10]. The T3-treated HKC constructs (Figure 2C), however, showed the highest density of collagen fibrils and ECM alignment out of all three conditions. Similar results were observed with HCFs (data not shown) [14], where T3 stimulation resulted in highly organized and denser ECM. 

### 3.3. Specific ECM markers Expression

#### 3.3.1. Type I and Type V Collagen (Col I and Col V)

**Figure 3:**
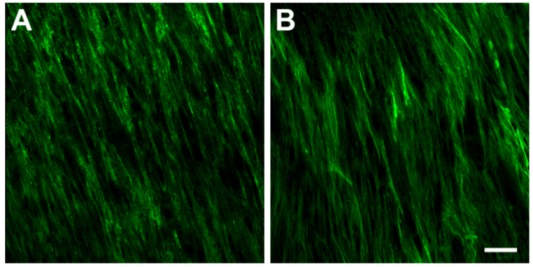
Representative confocal images of (**A**) Col I; and (**B**) Col V localization in TGF-β3-treated HKC constructs at 4 weeks. Note that the expression of both Col I and Col V remained unchanged under all conditions. Bar = 50 microns

Col I and V are considered vital collagens for the integrity and structure of the corneal tissue. Both Col I (Figure 3A) and Col V (Figure 3B) were found to be present in all the constructs and were not dependent upon the condition or cell type. 

#### 3.3.2. Type III Collagen (Col III)

**Figure 4:**
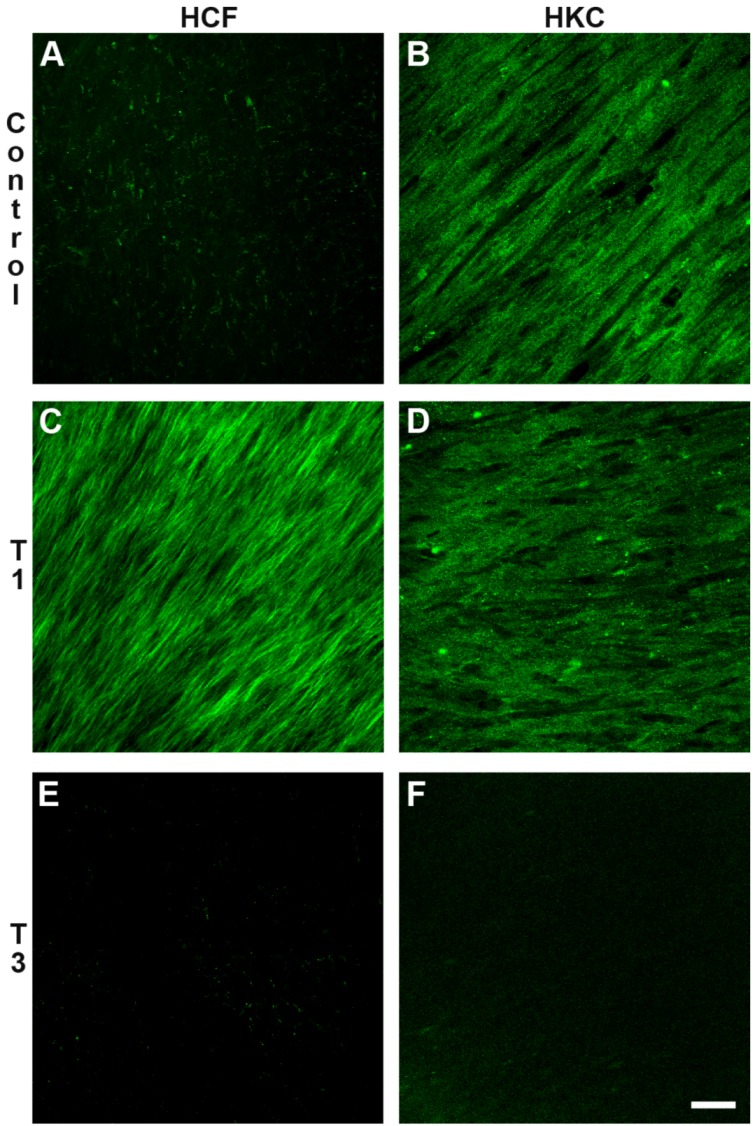
Confocal images of the immunolocalization of Col III in constructs at 4 weeks. (**A**) HCF treated with VitC only (Control); (**B**) HKC Control; (**C**) HCF treated with TGF-β1 (T1); (**D**) HKC with T1; (**E**) HCF treated with TGF-β3 (T3); (**F**) HKC with T3. Note that little, if any Col III was present in HCF Control (A), which is similar to that seen in a normal healthy human cornea; however, upon T1 treatment, Col III was upregulated (C). On the other hand, Col III was present in the HKC Control (B) and the localization did not change upon T1 treatment (D). Interestingly, the expression of Col III was significantly decreased for both cell types when T3 was introduced (E and F). Bar = 50 microns.

Col III is considered a marker for corneal fibrosis and scarring. Its expression is not normally present in healthy human corneas. As seen in Figure 4, the HCFs expressed little to no Col III if left untreated (Figure 4A); however, upon T1 treatment (Figure 4C), Col III expression was massively upregulated [10]. Unlike HCF, Col III expression was present in both Control (Figure 4B) and T1-treated (Figure 4D) HKC constructs. This data indicates that there is a difference between HCFs and HKCs. When constructs were stimulated with T3, neither cell type expressed Col III [14].

#### 3.3.3. Smooth Muscle Actin (SMA)

**Figure 5:**
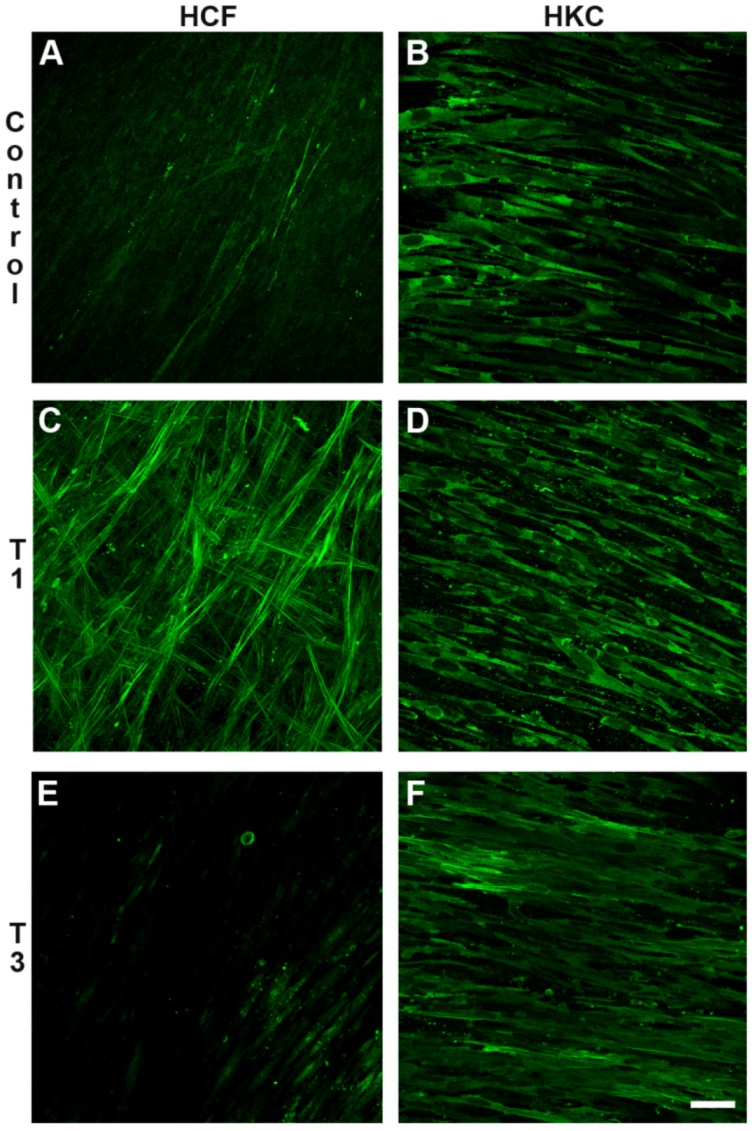
Confocal images of the immunolocalization of α-smooth muscle actin (SMA) in constructs at 4 weeks. (**A**) HCF treated with VitC only (Control); (**B**) HKC Control; (**C**) HCF treated with TGF-β1 (T1); (**D**) HKC with T1; (**E**) HCF treated with TGF-β3 (T3); (**F**) HKC with T3. The expression of SMA remained unchanged under all conditions in the HKC constructs (B, D and F); however, SMA was upregulated in the HCF constructs upon T1 stimulation (C) and downregulated upon T3 stimulation (E). Bar = 50 microns.

SMA is a marker of myofibroblasts, which are differentiated fibroblasts and are present in fibrotic stroma. As with Col III, SMA is not normally present in healthy human corneas. Also as with Col III, SMA expression in HCF increased with T1 treatment (Figure 5C) as compared with Control (Figure 5A) [10], and SMA localization was present at high levels in both Control (Figure 5B) and T1-treated (Figure 5D) HKC constructs. Again, this indicates a difference between the HCFs and HKCs. T3 treatment [14] minimized the expression of SMA in HCFs (Figure 5E); however, it had no effect on HKCs (Figure 5F), where the SMA expression remained high.

#### 3.3.4. Fibronectin (EDA-Fn)

**Figure 6:**
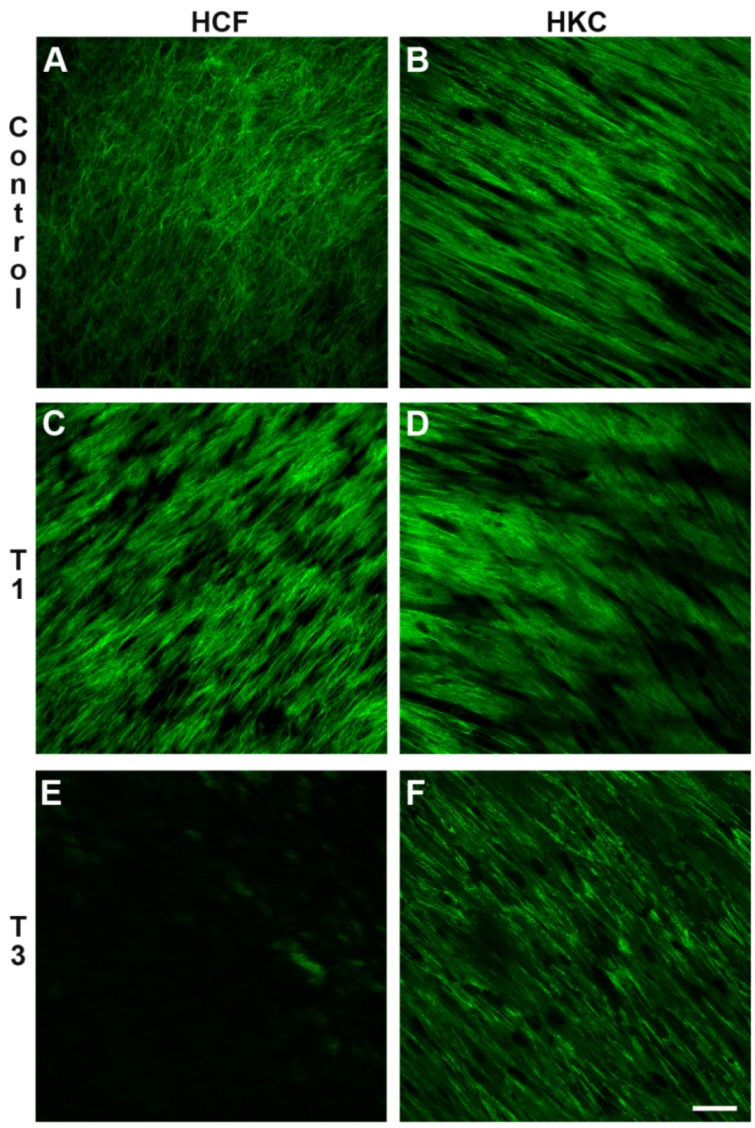
Confocal images of the immunolocalization of EDA-Fn in constructs at 4 weeks. (**A**) HCF treated with VitC only (Control); (**B**) HKC Control; (**C**) HCF treated with TGF-β1 (T1); (**D**) HKC with T1; (**E**) HCF treated with TGF-β3 (T3); (**F**) HKC with T3. The expression and alignment of EDA-Fn remained unchanged under all conditions in HKC (B, D and F). HCFs, however, showed an increase in alignment upon T1 treatment (C) and a significant downregulation of EDA-Fn upon T3 stimulation (E). Bar = 50 microns.

EDA-Fn is a glycoprotein of the ECM and is known to play a major role in wound healing and embryonic development [16]. As shown in Figure 6, both cell types secreted copious amounts of EDA-Fn under control conditions (Figure 6A,B). Upon T1 treatment of both cell types (Figure 6C,D), no significant difference in amounts of EDA-Fn was observed; however, with T3 stimulation, EDA-Fn expression was significantly reduced in the HCF constructs (Figure 6E), but the HKC constructs (Figure 6F) remained unaffected. Interestingly, the HKC constructs showed high alignment of EDA-Fn under all conditions (Figure 6B, D and F); whereas, the EDA-Fn alignment was only present in the HCF constructs after T1 treatment (Figure 6C). 

### 3.4. Fast Fourier Transform Analysis

**Figure 7:**
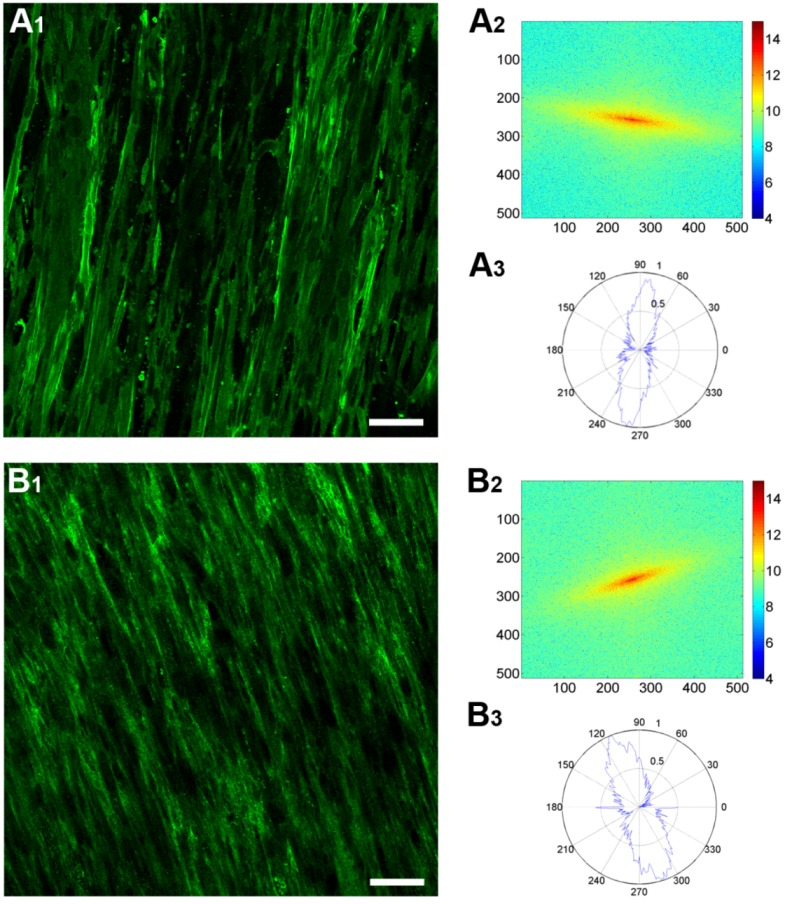
Representative images and plots for (**A**) cells; and (**B**) ECM for the Fast Fourier transform (FFT) analysis of the constructs at 4 weeks. (**A_1_**) Immunolocalization of anti-SMA (cells); (**A_2_**) Global orientation of the cells as indicated by 2D FFT transform; (**A_3_**) Polar histograms used to quantitate the orientation of the cells; (**B_1_**) Immunolocalization of anti-Col I (ECM); (**B_2_**) Global orientation of the ECM as indicated by 2D FFT transform; (**B_3_**) Polar histograms to quantitate the orientation of the ECM. Bar in A_1_ and B_1_ = 50 microns.

Z-series scans obtained with the confocal microscope were imported into a custom-made MATLAB program to calculate and analyze the cell and ECM alignment and orientation for each Z-scan based on FFT analysis. Figure 7 shows representative images used to analyze the data for both cell and ECM orientation. Figure 7A_1_ and B_1_ are single plane-of-focus (Z-scan) confocal images showing the cell (SMA) and ECM (Type I collagen) staining, respectively. The corresponding FFT analysis is also shown (Figure 7A_2,3_ and B_2,3_). The global orientation is indicated by the 2D FFT transform of the stained cells (Figure 7A_2_) or ECM (Figure 7B_2_). To quantify the orientation of the cells or ECM, we plotted polar graphs (or histograms), which indicate the intensity of the FFT transform images at different angles (Figure 7A_3_ and B_3_). The blue line on the polar graph equals the angle (θ) of the cells or ECM and shows the normalized integral intensity of all the pixels in the 2D FFT image within (0,θ)—the origin of the image—to (200,θ) polar coordinates. The dominant orientation corresponds to the peaks of the polar graph. Furthermore, from the same polar graph, we can calculate how aligned the overall population was by measuring the width of the peaks—the wider the peaks, the more dispersed the distribution of orientations and the more random the alignment. 

### 3.5. Cellular Alignment and Rotation

**Figure 8:**
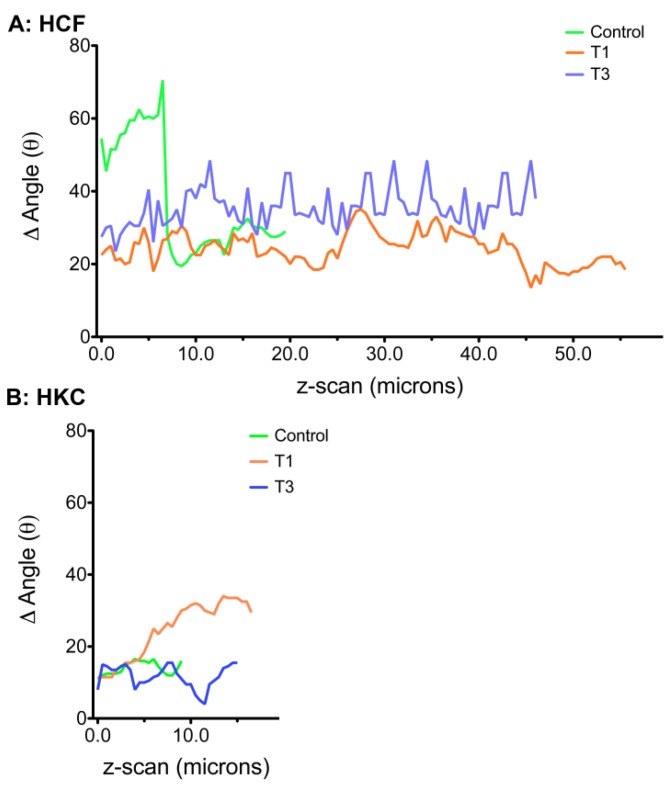
FFT quantification of cell alignment for both (**A**) HCFs; and (**B**) HKCs under all three conditions: Controls, TGF-β1 (T1), and TGF-β3 (T3).

Using the tools shown above, we quantified the cell orientation for each z-scan under all the conditions using constructs stained with anti-SMA. For analyzing the alignment, we plotted the width of the peak of the polar graph, or the Δ of angle, *versus* the z-scan position starting at 0 (top of the construct). The step size between z-scans was 0.5 microns. Figure 8 shows the quantification of the cell alignment for both HCFs (Figure 8A) and HKCs (Figure 8B). The lower the value on the Y-axis, the smaller the Δ of angle, thus the more aligned the cells. As seen in Figure 8A, HCFs showed higher cell alignment when stimulated with T1 and T3 when compared to Controls. Controls mainly aligned towards the bottom half of the construct, as indicated by the sudden drop in angle value. On the other hand, in Figure 8B HKCs showed high cellular alignment under all conditions, even under Controls, with T3 showing a trend for the highest alignment between the three conditions. Notably, HKCs were more aligned when compared to HCFs. 

**Figure 9:**
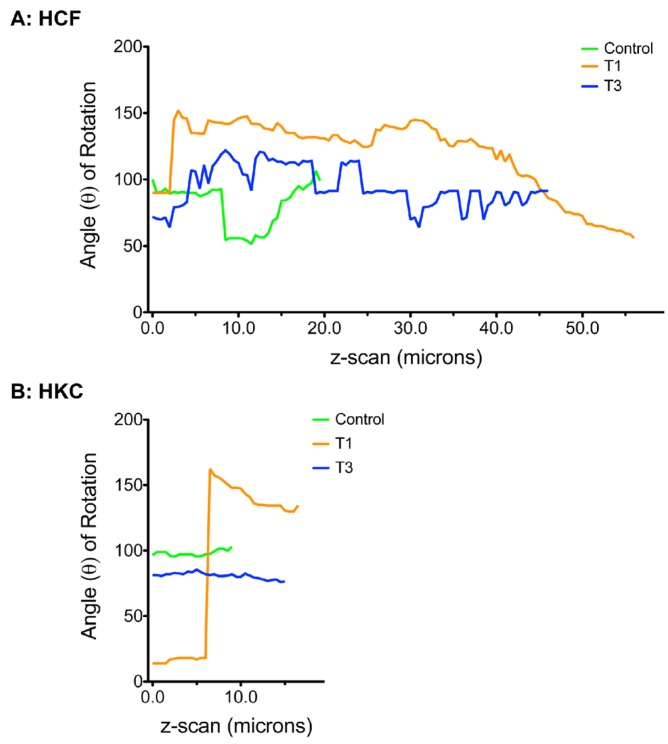
FFT quantification of cell rotation for both (**A**) HCFs; and (**B**) HKCs under all three conditions: Controls, TGF-β1 (T1), and TGF-β3 (T3).

In terms of cell rotation, the mean angle on the polar graph, which is equal to the direction that the cells are oriented, was calculated for each z-scan within a construct. The angles for each of the z-scans were then plotted on a graph *versus* the z-scan position, thus showing the rotation of the oriented cells. Therefore, the wider the range of the angles for a construct, the more cellular rotation is observed. In Figure 9, the cell rotation graphs for both cell types under all conditions are shown. HCFs (Figure 9A) showed cell rotation under all conditions. Controls had a range of ~40°, whereas T1 and T3 had a range of ~55°. HKCs (Figure 9B), on the other hand, showed minimal cell rotation in Control and T3-treated cells; however, T1-treated cells showed a single massive sudden cell rotation of ~110 °C. Overall, from the results in Figure 8 and Figure 9, HKCs were more aligned and had less rotation than HCFs. 

### 3.6. ECM Alignment and Rotation

**Figure 10:**
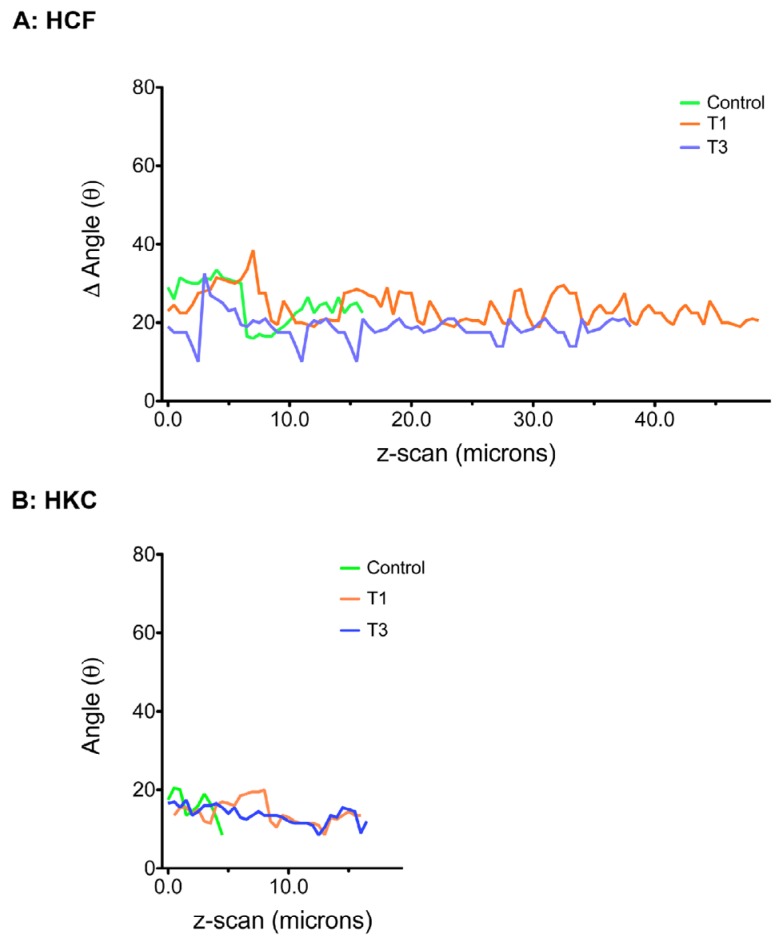
FFT quantification of ECM alignment for both (**A**) HCFs; and (**B**) HKCs under all three conditions: Controls, TGF-β1 (T1), and TGF-β3 (T3).

**Figure 11:**
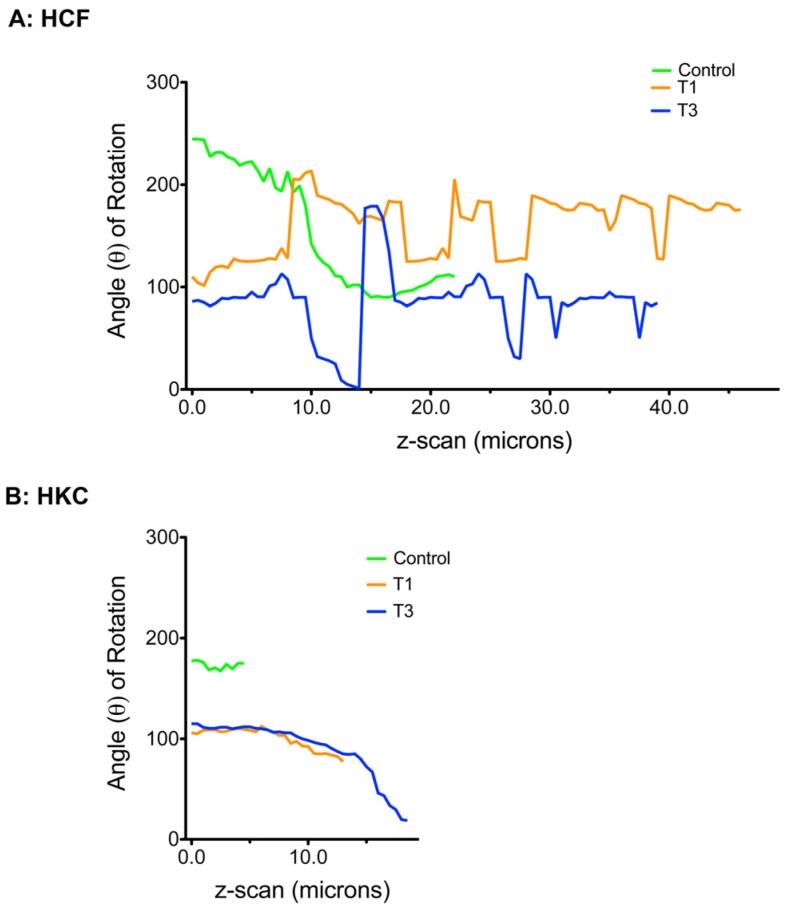
FFT quantification of ECM rotation for both (**A**) HCFs; and (**B**) HKCs under all three conditions: Controls, TGF-β1 (T1), and TGF-β3 (T3).

For ECM alignment and rotation (Figure 10 and Figure 11, respectively), we used the constructs stained with Type I collagen in order to quantify our data for all conditions. As can be seen in Figure 10A, HCFs secreted a relatively aligned ECM irrespective of the different treatments, with slightly less alignment in the Controls. With the HKCs (Figure 10B), the Controls only secreted a single layer of ECM, which appeared to be aligned (Figure 10B); however, T1 and T3 produced a 15–20 μm-thick ECM, which was equally aligned (Figure 10B). 

ECM rotation, and for this specific study, Col I rotation, was seen with both cell types (Figure 11). More specifically, HCFs (Figure 11A) showed ECM rotation throughout the constructs, under all treatments. T1 and T3 exhibited more frequent changes in rotation, whereas, the Controls had a smother rotation/transition. HKC Controls (Figure 11B) only produced a monolayer, therefore rotation could not be judged. T1 and T3 treatments, however, resulted in thicker ECM; therefore, the rotation of the ECM was more evident. For both T1 and T3, the rotation was smooth with no sudden changes. 

## 4. Discussion

Keratoconus is a degenerative disorder of the cornea where structural changes cause it to thin, thus reducing its strength, allowing the intraocular pressure to cause the cornea to protrude and assume a more conical shape [17]. Keratoconus affects one person in a thousand and can result in severe loss of vision and decreased quality of life [3]. The exact cause of Keratoconus is uncertain, and both environmental and genetic factors have been suggested; however, the findings remain inconclusive. This study’s aim was to establish and characterize a 3D *in vitro* model using human Keratoconus stromal cells (HKCs), and to determine if stimulation with two different isoforms of TGF-β can stimulate the generation of a more normal corneal stroma. 

Two of the main characteristics of the Keratoconus disease are (1) thinning of stromal ECM; and (2) scarring and/or opacity in the cornea. The pathogenetical process is still unclear; however, it is certain that the damage caused within the cornea results from the reduction in the cornea’s thickness and ultimately its biomechanical strength. In this study, we investigated the use of two TGF-β isoforms as ways to alter these defects *in vitro*. To-date, very few studies have examined growth factors/cytokines in Keratoconus, and in fact, in human anterior segment diseases [18]. Zhou *et al*. [19] found elevated levels of TGF-β and IL-1 in Keratoconus corneas; however, this is true for a variety of diseases, and therefore, not specific to Keratoconus. In our study, when TGF-β was used, we showed that HKCs were stimulated and showed abilities to generate an ECM that was similar to the one generated by HCFs. In fact, the ECM amounts produced were significantly higher when compared to cells without the TGF-β-isoform stimuli. This data indicates that the cells can be stimulated in a manner that can help provide a solution to the corneal thinning aspect of the disease. Since there are no animal models available, the significance of our findings becomes increasingly important. In future studies, we will investigate the effect of cellular contraction and cell generated forces that might lead to ECM thinning. However, in this study, we did not observe any obvious amounts of contraction. 

The role of collagen in healthy, as well as injured corneas is well established. Different collagen Types can be the difference between scarring and scar-free corneal healing. In Keratoconus disease, the occurrence of scarring in the cornea is a common problem and often leads to vision problems. Collagens type I, III, IV, V, VI, VII, and VIII are scattered throughout different corneal layers, as outlined by Rabinowitz in 1998 [4]. It is easily understood that any of these collagen types may be a candidate and responsible for the Keratoconus defects. Since the probable area of dysfunction in a Keratoconic patient is the stroma, it is easily understood that Type I, III and V collagen are strong candidates. In our previous studies, we have shown that when T3 was used to stimulate HCFs, there was a significant decrease, or even elimination, of specific fibrotic markers, such as Type III collagen [20,21,22], which has been routinely linked to fibrosis and scarring [20,21,22,23,24]. When T3 was used to stimulate HKCs, a similar effect was shown. This is very important considering that one of the problems with Keratoconus is corneal scarring and haze. In this study, T3 appears to stimulate the HKCs to produce more collagenous ECM with minimal fibrotic characteristics, which can be of great importance in an attempt to characterize and understand cellular progression of the Keratoconus disease.

## 5. Conclusions

We have developed a novel *in vitro* 3D model with human Keratoconus cells which allows for the study of cell stratification and matrix assembly and resembles the processes observed in Keratoconus disease.
